# Immunolocation and enzyme activity analysis of *Cryptosporidium parvum* enolase

**DOI:** 10.1186/s13071-017-2200-y

**Published:** 2017-05-31

**Authors:** Rongsheng Mi, Xiaojiao Yang, Yan Huang, Long Cheng, Ke Lu, Xiangan Han, Zhaoguo Chen

**Affiliations:** 1 State Key Laboratory of Veterinary Etiological Biology, Lanzhou, 730046 China; 2 Key Laboratory of Animal Parasitology of Ministry of Agriculture, Laboratory of Quality and Safety Risk Assessment for Animal Products on Biohazards (Shanghai) of Ministry of Agriculture, Shanghai Veterinary Research Institute, Chinese Academy of Agricultural Sciences, Shanghai, 200241 China; 3 Jiangsu Co-innovation Center for Prevention and Control of Important Animal Infectious Diseases and Zoonoses, Yangzhou, 225009 China

**Keywords:** *Cryptosporidium parvum*, Enolase, Immunolocation, Enzyme activity, Binding activity

## Abstract

**Background:**

Enolase is an essential multifunctional glycolytic enzyme that is involved in many biological processes of apicomplexan protozoa, such as adhesion and invasion. However, the characteristics of enolase in *Cryptosporidium parvum*, including the location on the oocyst and the enzyme activity, remain unclear.

**Methods:**

The *C. parvum* enolase gene (*cpeno*) was amplified by RT-PCR and sequenced. The deduced amino acid sequence was analysed by bioinformatics software. The gene was expressed in *Escherichia coli* BL21 (DE3) and purified recombinant protein was used for enzyme activity analysis, binding experiments and antibody preparation. The localisation of enolase on oocysts was examined *via* immunofluorescence techniques.

**Results:**

A 1,350 bp DNA sequence was amplified from cDNA taken from *C. parvum* oocysts. The deduced amino acids sequence of *C. parvum* enolase (CpEno) had 82.1% homology with *Cryptosporidium muris* enolase, and 54.7–68.0% homology with others selected species. Western blot analysis indicated that recombinant *C. parvum* enolase (rCpEno) could be recognised by *C. parvum-*infected cattle sera. Immunolocalization testing showed that CpEno was found to locate mainly on the surface of oocysts. The enzyme activity was 33.5 U/mg, and the Michaelis constant (*K*_*m*_) was 0.571 mM/l. Kinetic measurements revealed that the most suitable pH value was 7.0–7.5, and there were only minor effects on the activity of rCpEno with a change in the reaction temperature. The enzyme activity decreased when the Ca^2+^, K^+^, Mg^2+^ and Na^+^ concentrations of the reaction solution increased. The binding assays demonstrated that rCpEno could bind to human plasminogen.

**Conclusion:**

This study is the first report of immunolocation, binding activity and enzyme characteristics of CpEno. The results of this study suggest that the surface-associated CpEno not only functions as a glycolytic enzyme but may also participate in attachment and invasion process of the parasite.

**Electronic supplementary material:**

The online version of this article (doi:10.1186/s13071-017-2200-y) contains supplementary material, which is available to authorized users.

## Background

*Cryptosporidium* is an important zoonotic protozoan with a wide range of hosts, including humans and various animals. The main symptoms of cryptosporidiosis are diarrhoea, which can be fatal [1]. *Cryptosporidium* is the second most serious diarrheal disease in infants and young children in developing countries after rotavirus [2]. According to the World Health Organization, there are nearly eight million annual deaths of children under 5 years of age caused by diarrhoea [3].

Currently, no effective vaccine or drug has been found to prevent this disease, so it is important to identify specific target antigens and better understand the host immune response to the parasite. Nowadays, many moonlighting proteins in *Cryptosporidium* have been found to play an important role in parasite adhesion and invasion. For example, *C. parvum* elongation factor 1α (EF-1α), a novel protein associated with host cell invasion could be a candidate vaccine antigen against cryptosporidiosis [4]. *Cryptosporidium parvum* Clec (*Cp*Clec), a novel mucin-like glycoprotein, contains a C-type lectin domain (CTLD) and localised to the surface of the apical region, maybe playing a significant role in *Cryptosporidium*-host cell interactions [5].

Unlike other apicomplexan parasites, such as *Plasmodium falciparum* and *Toxoplasma gondii*, *Cryptosporidium* lacks many of the pharmacological targets, and no mitochondrial genome has been found [6]. Energy metabolism is a necessary process of biological survival, and the main way to obtain energy in the majority of higher organisms is the tricarboxylic acid (TCA) cycle and β-oxidation process [7]. However, according to the complete genome sequence, these metabolic pathways are absent in *C. parvum* [8]. Therefore, the main energy pathway in *C. parvum* is probably glycolysis [9], so enzymes involved in the glycolytic pathway may be potential targets for therapeutic agents.

As a key enzyme in the glycolytic pathway, enolase can catalyse the reversible interconversion of 2-phosphoglycerate (2-PGA) and phosphoenolpyruvate (PEP), which exist in many species. Many studies have clarified that enolase is a highly conserved and multifunctional protein in both prokaryotes and eukaryotes [10]. It has been localised in the cytoplasm, cell surface and nucleus of various mammalian cells [11]. According to previous reports, enolase has many moonlighting functions [12–19]. For example, enolase can be displayed on the surface of several kinds of cells including certain tumour cells and act as a plasminogen binding receptor, which promotes tissue invasion and dissemination through the body [12–17]. Enolase in the RNA degradosome plays a crucial role in the rapid decay of glucose transporter mRNA in response to phosphosugar stress in *Escherichia coli* [18]. In *Arabidopsis thaliana*, enolase can bind to the zinc finger protein gene and impair cold-responsive gene transcription [19]. Nuclear regulatory functions exist in the *T. gondii* enolase that may involve changes in chromatin structure [20]. In *P. falciparum*, biliverdin (BV) can target enolase and eukaryotic initiation factor (eIF2α) to reduce the intraerythrocytic development of the parasite [21]. In *Cryptosporidium* spp., several sequences of enolase genes have been published, including that for *C. hominis* (GenBank XM_663094), *C. muris* (GenBank NW_002196570) and *C. parvum* (GenBank XM_626138). However, to our knowledge, until now there was no related report concerning the characteristics and functions of enolase in *Cryptosporidium* spp. The aim of the present study was to investigate the localisation, binding activity, enzymatic activity and factors that may influence the activity of enolase in *C. parvum*.

## Methods

### Parasite multiplication and purification

The Iowa isolate oocysts of *C. parvum* were purchased from Waterborne Inc. (New Orleans, LA, USA) and multiplied in newborn Holstein bull calves in our laboratory. Oocysts from the faeces were concentrated with Sheather’s sucrose flotation as previously described by Chen et al. [22] and purified by discontinuous sucrose gradients [23]. All the purified oocysts were stored in 2.5% potassium dichromate (K_2_Cr_2_O_7_) solution at 4 °C and used for RNA extraction or immunofluorescence assay within 1 month.

### Gene amplification, sequence and phylogenetic analysis

Total RNA was extracted from freshly isolated oocysts of *C. parvum* using TRIzol Reagent (Invitrogen, Carlsbad, CA, USA), and precipitated with isopropanol, rinsed with 70% ethanol, then finally dissolved in 20 μl of RNase-free water. RNA integrity was checked in 1.0% agarose gels and concentration was tested by NanoDrop 2000C (Thermo Fisher Scientific, Carlsbad, CA, USA) by reading extinction values at 260/280 nm. The total RNA was reversely transcribed into cDNA by a PrimeScript™ II Reverse Transcriptase kit according to the manufacture’s protocol (Takara, Dalian, China).

The *C. parvum* enolase (CpEno) encoding gene (*cpeno*) was amplified by polymerase chain reaction (PCR) using a forward primer 5′-GGA ATT CAG TTT AGC CAT GCC TTC TAT ATT GTC-3′ and the reverse primer 5′-CCC AAG CTT TTA TCT GAT TCT TGC GAT GGG TTT TC-3′ which introduced restriction endonuclease sites *Eco*R I and *Hin*d III (underlined), respectively. PCR was performed using the following conditions: an initial denaturation at 94 °C for 5 min and then 30 cycles of 45 s at 94 °C, 45 s at 55 °C and 1 min at 72 °C, followed by 7 min at 72 °C. The PCR products were analyzed on a 1.2% agarose gel and purified from the gel using AxyPrep^TM^ DNA Gel Extraction Kit (Axygen, Suzhou, China), then cloned into pMD 18-T vector (Takara) and sequenced using ABI 3730xl DNA Analyzer (Applied Biosystems, Foster City, USA) repeatedly. The sequence was analysed by the BLAST (http://blast.ncbi.nlm.nih.gov/Blast.cgi) program for homology search, and the predicted amino acid sequence was compared with reported enolase sequences from a wide range of species. Additionally, the conserved motifs, signal peptides, transmembrane regions and glycosyl-phosphatidyl anchor sites of the enolase sequence were analysed by the online resources, including NCBI conserved domains (http://www.ncbi.nlm.nih.gov/Structure/cdd/wrpsb.cgi), SignalP 4.1 Server (http://www.cbs.dtu.dk/services/SignalP), TMHMM Server v. 2.0 (http://www.cbs.dtu.dk/services/TMHMM-2.0/), and GPI-SOM (http://gpi.unibe.ch/) [24, 25]. The phylogenetic evolutionary analysis of amino acid sequences of CpEno and enolases of other organisms, such as flagellate protozoa, nematodes, tapeworms, flukes, bacteria, rabbit and human, were determined using MEGA 7.0 software [26].

### Expression and purification of recombinant CpEno (rCpEno)

The *cpeno* gene was digested from recombinant plasmids pMD18T-*cpeno* by *Eco*RI and *Hin*dIII and inserted into the prokaryotic expression vector pET-28a (+) (Novagen, La Jolla, CA, USA). Recombinant plasmid pET28a-*cpeno* was transformed into BL21 (DE3) competent cells, and the transformed bacteria were induced using isopropyl-β-D-1-thiogalactopyranoside (IPTG) at a final concentration of 1 mM for 6 h at 37 °C. The rCpEno was purified under native conditions by Ni-NTA His Bind Resin affinity chromatography (Novagen) according to the manufacturer’s instructions. The purified rCpEno was resolved on 12% sodium dodecyl sulfate-polyacrylamide gel electrophoresis (SDS-PAGE) and quantified by Pierce™ BCA Protein Assay Kit (Pierce, Rockford, lL, USA).

### Western blot analysis

For Western blot analysis, the purified rCpEno was separated on 12% SDS-PAGE and electrotransferred to a PVDF membrane (Merck Millipore, Billerica, MA, USA) using a Trans-Blot® SD Semi-Dry Transfer Cell (Bio-Rad, Hercules, CA, USA) according to standard procedures. Induced pET28a (+) plasmid transformed BL21 (DE3) competent cells were selected as the control. The membranes were blocked with 5% skim milk (Oxoid, Basingstoke, Hants, UK) for 2 h at room temperature and incubated with His-Tag mouse mAb (1:1,000) (Cell Signaling Technology, Danvers, MA, USA) or serum isolated from cattle artificially infected with *C. parvum* (1:200) for 1 h, followed by incubation with anti-mouse IgG (whole molecule)-Peroxidase conjugate antibody (Sigma-Aldrich, Louis, MO, USA) or anti-bovine IgG (Sigma-Aldrich) at 1:2,000 dilution for 1 h at room temperature. Reaction bands were detected using a DAB Substrate Kit (Pierce).

### Polyclonal antibody production

New Zealand rabbits were purchased from Slack Laboratory Animal Co., Ltd., (Shanghai, China). Before immunisation, faeces were collected from the rabbits for 3 days to check for *Cryptosporidium* spp. infection. One millilitre of purified rCpEno (1 mg/ml) emulsified with the same dose of Freund’s complete adjuvant (Sigma-Aldrich) was injected once into the *Cryptosporidium* spp. free rabbits, and then 1 ml rCpEno (1 mg/ml) emulsified with Freund’s incomplete adjuvant (Sigma-Aldrich) was injected the next two times at 2-week intervals. Blood samples were collected, and the titers of rabbit antiserum against rCpEno were evaluated using enzyme-linked immunosorbent assay (ELISA). ELISA plate wells were coated with purified rCpEno (2 μg/ml) and incubated overnight at 4 °C. Rabbit muscle enolase (Sigma-Aldrich) was used as a negative control in this experiment. Then the plate wells were blocked with skim milk in PBST (Tween 0.05%) at 37 °C for 1 h. Rabbit anti-*C. parvum* enolase antibody with different concentrations were added to the wells and finally incubated with horseradish peroxidase (HRP) conjugated anti-rabbit IgG antisera (Sigma-Aldrich). Reactions were developed with tetramethylbenzidine (TMB) substrate solution (Sigma-Aldrich) and terminated with 2 M H_2_SO_4_. The values of OD450 were read by an automated ELISA reader (BioTek, Winooski, VT, USA).

### Protein immunolocalization in oocysts

Immunolocalization of enolase in *C. parvum* oocysts was analysed as previously described by Paluszynski et al. [27] with minor modifications. Briefly, 50 μl of the purified oocysts (approximately 10^5^ oocysts/ml) were added to pre-cleaned glass slides and air-dried. Then, the slides were fixed with 4% absolute paraformaldehyde in PBS for 15 min at room temperature. Oocysts were permeabilized with 1% Triton X-100 in 1% bovine serum albumin (BSA) for 15 min and blocked with 1% BSA overnight. The slides were incubated with polyclonal antibodies specific for rCpEno (1:100 dilution in 1% BSA) and washed three times in PBS. Negative controls were treated in the same way but using BSA and pre-immunization antisera. Fluorescein isothiocyanate (FITC)-conjugated goat anti-rabbit IgG as secondary antibody (1:1,000 dilution in 1% BSA) (Life Technologies, NY, USA) was added to the slides and incubated for 1 h at 37 °C. After washing three times with PBS, 4’,6’-diamidino-2-phenylindole (DAPI) (Life Technologies) was used to label nuclei for 15 min at 4 °C. The slides were observed by a Nikon Eclipse 80i fluorescence microscope with a DS-Fi1 colour cooled digital camera (Nikon, Tokyo, Japan).

### Enzyme activity and kinetic assay

The enzyme activities of rCpEno were determined by measuring the conversion of 2-PGA (Sigma-Aldrich) to PEP (Sigma-Aldrich) at 25 °C, pH 7.4 as described by Han et al. [28] with minor modifications. In brief, 1 mM 2-PGA was added to a 2 ml reaction system, and 20 μg rCpEno was added to the reaction buffer. PEP release values were monitored by NanoDrop 2000C at 240 nm for 40 s intervals in 10 min. As the experiment control, rabbit muscle enolase (positive control) and BSA (negative control) were used in place of rCpEno for the assay.

For the enzyme kinetics research of rCpEno, different concentrations of 2-PGA (0.125, 0.25, 0.5, 1.0, 1.5, 1.75 and 2.0 mM) were added to the reaction buffer. Then, 20 μg rCpEno was added for the assay. The production of PEP was tested at an absorbance value of 240 nm at 1 min intervals for 10 min. The Michaelis constant (*K*_*m*_) and the maximum reaction velocity (*V*_*max*_) for rCpEno were determined from double-reciprocal Lineweaver-Burk plots.

### Effects of pH, temperature and metal ions on enolase activity

The effects of external factors on the enzyme activity of rCpEno were tested under the following conditions. For the power of hydrogen effects, different pH values (5.5, 6.0, 6.5, 7.0, 7.5, 8.0 and 8.5) were used for the assay at room temperature. To examine the effect of reaction temperature, rCpEno activity was tested at 4 °C, 22 °C, 37 °C and 46 °C at pH 7.4. For the metal ions influence, different concentrations of Ca^2+^ (0, 20, 40, 80 and 160 mM), K^+^ (0, 100, 200, 300 and 400 mM), Mg^2+^ (0, 25, 50, 100 and 200 mM) and Na^+^ (0, 50, 100, 150, 200, 250 and 300 mM) were measured at 25 °C, pH 7.4. Enolase activities in each of the above tests were compared under the same condition involving 1.0 mM 2-PGA substrate and 20 μg of rCpEno in a 2 ml reaction system. All experiments were repeated three times.

### Binding activity of rCpEno to human plasminogen

ELISA plate binding assay was used to determine the binding activity of rCpEno to human plasminogen as described by Bao et al. [16]. Briefly, 10 μg/well of purified rCpEno was added into 96-well plate and incubated at 4 °C overnight. BSA (10 μg/well) was used as a negative control. After being washed three times with PBST, the plate wells were blocked with 5% skim milk (Oxoid) in PBST at 37 °C for 2 h. After washing, different concentrations of human plasminogen (0, 1, 5, 10, 15, 20, 25 and 30 μg/ml in PBST) (Merck Millipore) were added to each well and incubated at 37 °C for 2 h. Then the plates were incubated with rabbit anti-plasminogen polyclonal antibody (100 μl/well, 1: 3,000) (Abcam, MA, USA) at 37 °C for 1.5 h. After washing three times with PBST, the plate wells were incubated with goat anti-rabbit IgG-HRP (100 μl/well, 1: 5,000) (Sigma-Aldrich) at 37 °C for 1 h. Finally, the colour reaction was performed by adding soluble TMB substrate solution and terminated with 2 M H_2_SO_4_. Absorbance at OD450 was read using a spectrophotometer (BioTek).

### Statistical analysis

Antibody levels and enolase activities were performed using GraphPad Prism 6.0 software for Windows (GraphPad Software, La Jolla, CA, USA), and the data in this study were the mean values ± standard deviations. The significance analysis of differences between 0 mM metal ions and other concentrations at the same time point were analysed by IBM SPSS Statistics V21.0 for Windows (International Business Machines Corp, New York, USA). The differences were considered significant when *P* < 0.05 by one-way ANOVA test.

## Results

### Sequence and phylogenetic analysis of CpEno

**Fig. 1:**
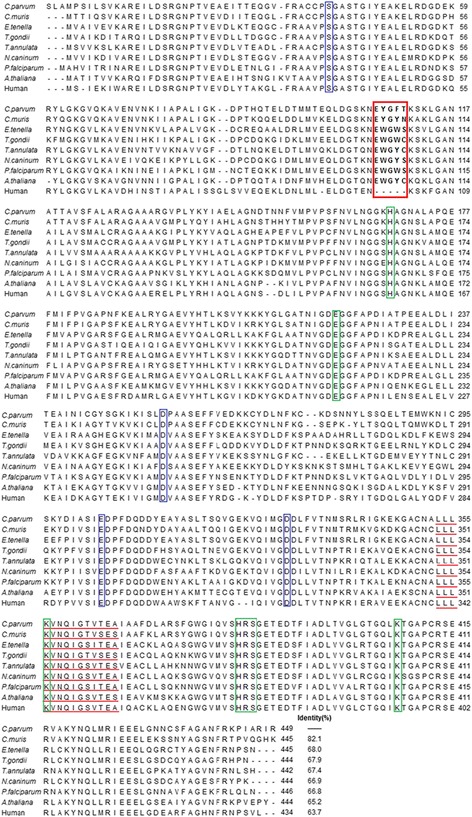
Alignment of amino acid sequence of CpEno and other species. The GenBank Accession nos. of these species are as follows: *C. parvum* (XM_626138), *C. muris* (XM_002139230), *E. tenella* (AF353515), *T. gondii* ENO2 (AY155668), *T. annulata* (HQ646253), *N. caninum* (XM_003883950), *P. falciparum* (U00152), *A. thaliana* (AF424603) and Human gamma-2 enolase (M22349). Similar to *A. thaliana*, a plant-like five-amino-acid sequence was present in apicomplexan parasites (shown in the *red box*) and was not found in the human sequence. The Mg^2+^ binding motifs are shown in a *blue box*, substrate-binding motifs are shown in a *green box*, plasminogen binding motifs are shown in a *black box*, and the enolase signature is underlined in *red*

With cDNA from *C. parvum* oocysts as the template, a 1,350 bp gene was amplified successfully as theoretically predicted (Additional file 1: Figure S1). BLAST analysis demonstrated that the sequence had 100% identity with the reported *C. parvum* Iowa II enolase gene sequence (GenBank XM_626138), and encoded a 449 amino acid protein with a theoretical molecular weight of 48.6 kDa. The CpEno amino acid sequence showed 82.1% homology with the *C. muris* enolase sequence (GenBank XM_002139230). The highest homology of amino acid sequences mainly came from apicomplexan protozoa, such as *Eimeria tenella*, *Theileria annulata*, *T. gondii*, *Neospora caninum* and *P. falciparum*, which exhibited 68.0, 67.9, 67.3, 66.9 and 66.8% similarities, respectively (Fig. 1).

CpEno also had a plant-like five-amino-acid sequence (EYGFT) between sites 108 to 112, which was similar to others apicomplexan parasites (Fig. 1). Using online server analysis, CpEno showed a high degree of conservation with other species, including full conservation of the Mg^2+^ binding motifs (S^41^, D^255^, E^304^ and D^331^), substrate-binding motifs (H^168^, E^220^, K^356^, ^384^HRS^386^ and K^407^) and the enolase signature (^353^LLLKVNQIGSVTEA^366^) (Fig. 1). Similar to the *Streptococcus pneumonia* enolase amino acid sequence (^248^FYDKERKVY^256^) [14], a plasminogen binding motif (^261^FFVEDKKCY^269^) was also found in CpEno and other species (Fig 1).

**Fig. 2:**
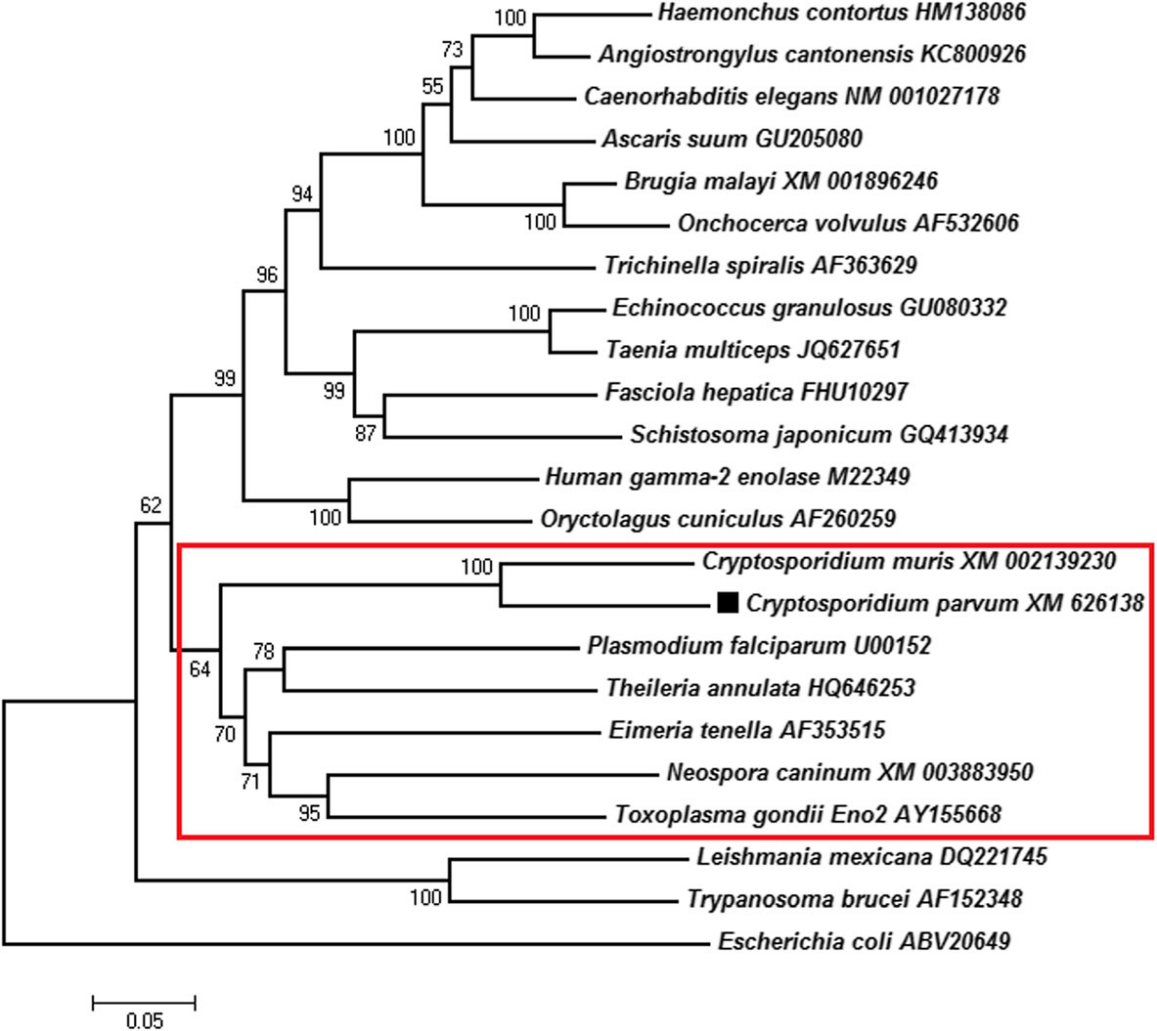
Phylogenetic analysis of CpEno with other enolase sequences. The phylogenetic tree was constructed using the neighbor-joining method by MEGA 7 software. The percentages of replicate trees in which the associated taxa clustered together in the bootstrap test (1,000 replicates) are shown next to the branches. The analysis involved 23 amino acid sequences, and all the apicomplexan protozoa located in the same branch (*red box*)

Phylogenetic analysis showed that the *C. parvum* enolase sequence was closest to *C. muris*, and then other apicomplexan protozoa. All of them located in the same branch. In all selected animals, CpEno had a closer relationship with nematodes, tapeworms, flukes and mammals (human and rabbit) than with flagellate protozoa and bacteria (Fig. 2).

### Expression and Western blot analysis

Using T4 DNA ligase (TaKaRa), *cpeno* was ligated into the pET-28a (+) vector and the recombinant plasmid was transformed into competent *E. coli* BL21 (DE3) to express the recombinant fusion protein after inducing with IPTG. The protein was analysed by SDS-PAGE and purified by Ni-NTA His Bind Resin affinity. Coomassie staining of the gels showed that most rCpEno remained within the supernatant (Additional file 2: Figure S2, Lanes 3 and 4), as well as a single band of 52.5 kDa protein, in agreement with the expected molecular mass including the 6 × His-tagged recombinant polypeptide (Additional file 2: Figure S2, Lanes 5 and 6). The recombinant protein was further characterised by Western blot detection with His-Tag mouse mAb and positive serum isolated from *Cryptosporidium* infected cattle (1:200) (Additional file 3: Figure S3). The results confirmed the recombinant protein could react with the two antibodies tested. In contrast, the induced pET28a (+) plasmid transformed *E. coli* BL21 (DE3) did not react with the positive serum or His-Tag mouse mAb.

### Anti-rCpEno polyclonal antibody preparation

**Fig. 3:**
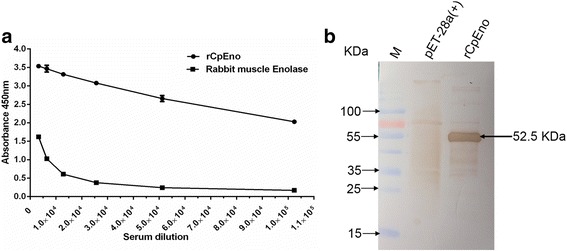
Polyclonal antibody detection of rCpEno immune serum. Rabbit polyclonal antisera were collected after the third immunisation. The reactivity and specificity of antibodies were tested by ELISA (**a**) and Western blot (**b**). A series of diluted antibodies were examined for their specific binding activity to either rCpEno or rabbit muscle enolase (negative control). A good antibody titer was detected when the dilution multiple was higher than 1/102,400, and a 52.5 kDa specific band was observed by western blot assay. Data shown are the mean values ± standard deviations

Purified rCpEno was used to generate rabbit polyclonal antisera. One week after the third immunisation, the reactivity and specificity of antibodies were tested by ELISA and western blot. The antibody titer of immune serum was more than 1/102,400. At that dilution, the optical density (OD) of immune sera was also higher than 2.0. While using rabbit muscle enolase as a negative control, no reaction was found when the dilution of immune serum was more than 1:6400 (Fig. 3a). A single band of about 52.5 kDa was recognised by immune serum using Western blot assay, and the induced pET28a (+) plasmid transformed *E. coli* BL21 (DE3) did not react with the immune serum (Fig. 3b).

### The localisation of CpEno in *C. parvum* oocyst

**Fig. 4:**
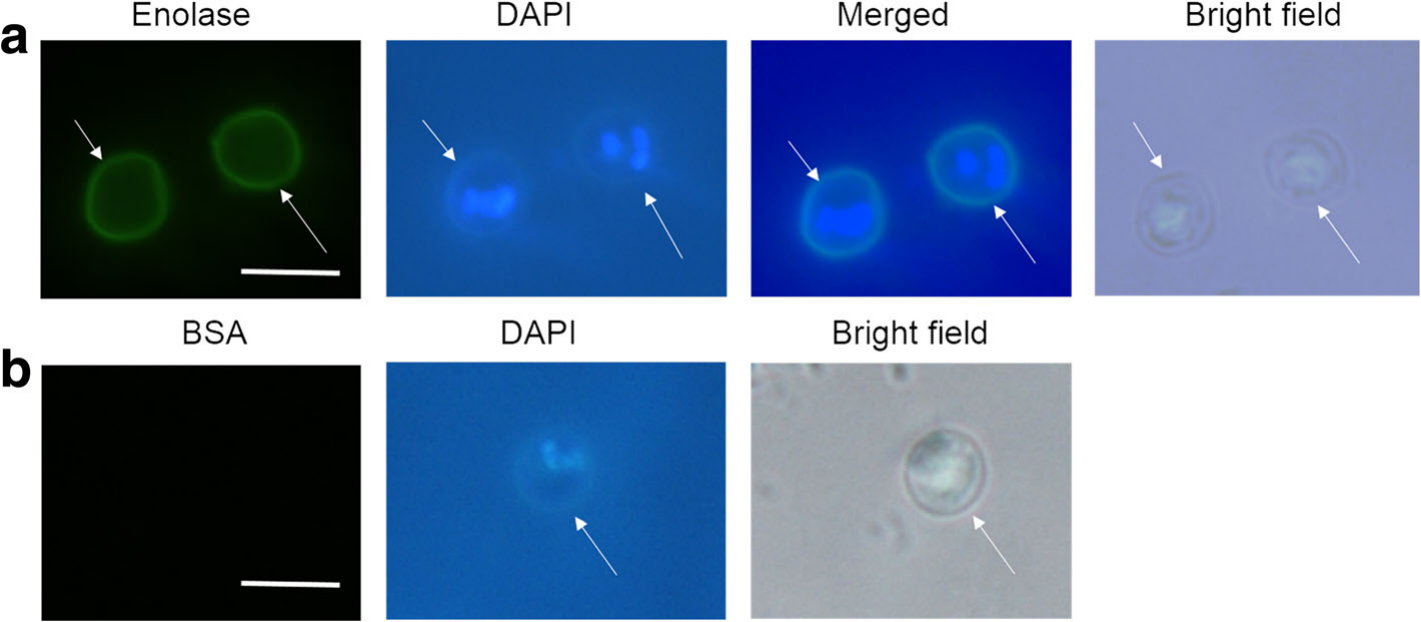
Immunolocalization of enolase in *C. parvum* oocysts. Oocysts were incubated with rabbit anti-enolase serum (**a**) and a BSA (negative control) (**b**). Enolases were located on the surface of oocysts (**a**), and no signals were found with BSA (**b**). Different fluorescent probes were used, rabbit anti-CpEno (*green*) and DAPI (nuclear marker) (*blue*). Arrows indicate *C. parvum* oocysts. *Scale-bars*: 5 μm

Freshly collected oocysts of *C. parvum* were fixed and treated with rabbit anti-rCpEno sera. The detected proteins were visualised by fluorescence microscopy using FITC-labelled secondary antisera against rabbit immunoglobulins. Clear signals were found surrounding the oocysts incubated with rabbit anti-rCpEno serum indicating that the protein localised on the surface (Fig. 4a). No signals were obtained after treatment of the slide with BSA (Fig. 4b) or pre-immunization antisera (data not shown).

### Enzyme activity and effects of pH, temperature and metal ions

**Fig. 5:**
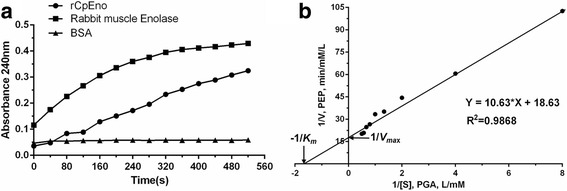
Enzymatic characterization of rCpEno. **a** Enzymatic activity of rCpEno was determined by measuring the conversion of 2-PGA to PEP. The absorbance value of rCpEno at 240 nm was lower than rabbit muscle enolase (positive control), and nearly no value was detected in BSA (negative control) at the same time point. **b** Enzyme kinetic research of rCpEno. The conversion rate (*V*) of 2-PGA (*S*) to PEP was measured at 240 nm using 20 μg of rCpEno and various concentrations of 2-PGA (0.125–2 mM). *K*_*m*_ and *V*_*max*_ for rCpEno were determined as 0.571 mM/l and 0.054 mM/l · min, respectively, using a Lineweaver-Burk double-reciprocal plot

The enzyme activity of rCpEno was detected by NanoDrop 2000C at 240 nm by increasing the amount of 2-PGA to PEP. The activity of rCpEno in catalysing the conversion of 2-PGA to PEP was lower than rabbit muscle enolase; however, almost no reaction was detected in BSA (Fig. 5a). Kinetic measurements using 2- PGA as the substrate gave a specific activity of 33.5 U/mg. A Michaelis-Menten plot was fitted to the equation *V* = *V*_*max*_ *S*/(*S* + *K*_*m*_), and the *K*_*m*_ for the forward reaction was 0.571 mM/l, the *V*_*max*_ was 0.054 mM/l · min (Fig. 5b).

**Fig. 6:**
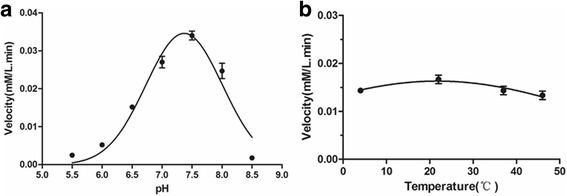
Effect of pH and temperature on the catalytic/enzymatic activity of rCpEno. Various pH (5.5–8.5) and temperature (4 °C, 22 °C, 37 °C and 46 °C) were determined to measure enzymatic activities using 20 μg of rCpEno at 240 nm. The maximal enzymatic reactivity of rCpEno occurred at pH 7.0 to 7.5 (**a**), and minor effects were found at temperatures from 4 °C to 46 °C (**b**)

**Fig. 7:**
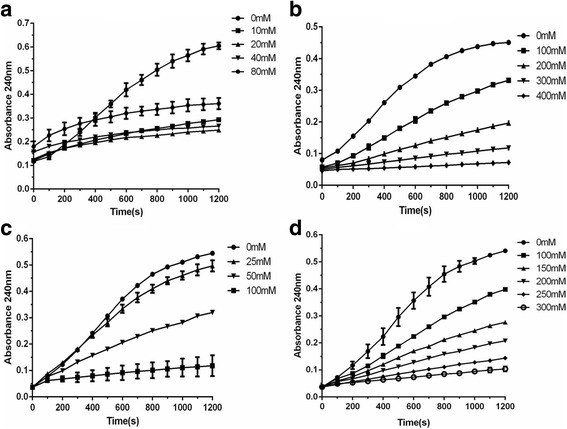
Effect of metal ions on the catalytic/enzymatic activity of rCpEno. Different concentrations of metal ions (Ca^2+^, K^+^, Mg^2+^ and Na^+^) (**a**, **b**, **c** and **d**) were measured at room temperature and pH 7.4. The reaction mixtures consisted of 20 μg purified rCpEno and one mM 2-PGA. The enzymatic activities of rCpEno were inhibited with increased concentrations of metal ions (*P* < 0.05). However, low concentrations of Mg^2+^ had almost no influence on enzyme activity (*P* > 0.05). The experiments were performed in triplicate, and the data shown are the mean values ± standard deviations

With regards to external influencing factors, pH 7.0 and pH 7.5 were found to be the most suitable pH value (Fig. 6a). Minor effects on the activity of rCpEno were found when the temperature changed from 4 °C to 46 °C (ANOVA: *F*_(3,8)_ = 3.273, *P* = 0.065) (Fig. 6b). The rCpEno activity was inhibited with increase of Ca^2+^(ANOVA: *F*_(5,72)_ = 18.719, *P* < 0.0001), K^+^(ANOVA: *F*_(4,60)_ = 21.980, *P* < 0.0001), Mg^2+^(ANOVA: *F*_(3,48)_ = 9.580, *P* < 0.0001) and Na^+^(ANOVA: *F*_(5,72)_ = 11.947, *P* < 0.0001) concentrations (Fig. 7), while low concentrations of Mg^2+^ (25 mM) had no obvious effect on enzyme activity (ANOVA: *F*_(1,24)_ = 0.163, *P* = 0.690) (Fig. 7c).

### Binding assay of rCpEno to human plasminogen

**Fig. 8:**
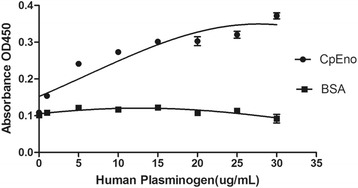
Binding activity of rCpEno to human plasminogen. The binding ability of rCpEno to human plasminogen was tested by ELISA. CpEno could bind to human plasminogen, and the binding activities of rCpEno were raised along with the concentrations of plasminogen increasing. BSA was not able to bind to human plasminogen

An ELISA experiment confirmed that rCpEno could bind to human plasminogen in a dose-dependent pattern. BSA showed no interaction with human plasminogen (Fig. 8).

## Discussion

*Cryptosporidium parvum* is one of the most common species in the genus to infect mammalian intestinal epithelial cells (IECs) with the onset of diarrhoea as a remarkable characteristic [29]. Genome sequencing and biochemical data have revealed that *C. parvum* lacks many mitochondrial proteins, including those required for the TCA cycle, fatty acid oxidation [8]. However, some essential metabolic pathways are still present in *C. parvum*. Glycolysis as the source of energy and carbon seems to play an important role in these pathways [30]. In addition to being an essential glycolytic enzyme, several studies have shown that enolase is a multifunctional protein, which participates in many important processes, such as motility, adhesion, invasion, differentiation, development and gene regulation in protozoa, including *E. tenella* [31, 32], *Entamoeba histolytica* [33], *Giardia lamblia* [34], *Leishmania donovani* [35], *Plasmodium* spp. [21, 36–48], *T. gondii* [20, 49, 50], *Trypanosoma* spp. [51] and *Trichomonas vaginalis* [52]. However, to our knowledge, the location and characteristics of *C. parvum* enolase are still unclear.

The complete encoding gene of CpEno was amplified from *C. parvum* cDNA and sequenced in this study. Sequence analysis showed that CpEno had 82.1% identity with *C. muris* enolase, a high degree of conservation with apicomplexan parasites (from 66.8 to 68.0% sequence identity), and 54.7 to 65.2% with other species. CpEno also has many conserved sites, such as metal- and substrate-binding motifs and an enolase signature which is similar to that in *Schistosoma bovis* and *Ornithodoros moubata* [53, 54], indicating that this protein is conserved in different phylum in the animal kingdom.

Sequence and phylogenetic analysis also showed that CpEno and other apicomplexan protozoa enolases were located in the same branch, and shared a plant-like pentapeptide insert (i.e. *C. parvum* ^106^EYGFT^110^, *C. muris* ^103^EYGYN^107^, *E. tenella* ^103^EWGWS^107^, *N. caninum* ^103^EWGYS^107^, *P. falciparum* ^104^EWGWS^108^, *T. annulata* ^103^EWGYC^107^, *T. gondii* ENO1 ^103^EWGYS^107^ and *T. gondii* ENO2 ^103^EWGWC^107^) [31, 55–59], which is more closely related to plant enolase (i.e. *A. thaliana* ^103^EWGYC^107^) than to the mammalian counterpart. Many studies have reported that this site is important for activities of enolases in apicomplexan protozoa. Vora et al. [39] found that deletion of this insert resulted in a 100-fold decrease of full enzyme activity and caused dissociation of the dimeric form into monomers in *P. falciparum*. A similar deletion provoked a decrease of substrate affinity in *T. gondii* ENO1 [57]. Recent studies showed that this motif could stabilise *P. falciparum* enolase in an active form and was also a protective antigenic epitope [46, 47]. Mukherjee et al. reported that a deletion variant Δ-rPfeno (*P. falciparum* enolase lacking insert sequence ^104^EWGWS^108^) was incapable of binding to PWWP (a conserved motif of the ookinete sequence) peptides, implying that this insert was involved in mosquito midgut invasion [48]. Because this unique region is absent in human enolase, it may be a potential drug target site for inhibitor design. However, the function of this insert in CpEno needs further studies.

According to the *C. parvum* genome sequence (http://cryptodb.org/cryptodb/), we found that CpEno was a single gene, which was similar to most studies of apicomplexan parasites, such as *E. tenella* [31], *Plasmodium* spp. [37, 43, 55], *T. annulata* [59], and was in contrast to studies of *T. gondii* enolase which reported two stage-specific glycolytic enolases, ENO1 and ENO2 [20, 56, 57].

In this study, the purified rCpEno could convert 2-PGA to PEP and the reaction activity was 33.5 U/mg, which was similar with *P. falciparum* (30 ± 3 U/mg) and *T. annulata* (40 U/mg) [59, 60]. Compared with other apicomplexan parasites, the *K*_*m*_ value of rCpEno (0.571 mM) was higher than in *P. falciparum* (0.041 ± 0.004 mM), *T. gondii* ENO1 (0.0768 ± 0.0064 mM), *T. gondii* ENO2 (0.0777 ± 0.0049 mM) and *T. annulata* (0.106 mM) [57, 59, 60].

With regards to environmental effect factors, rCpEno showed a stable temperature characteristic. Minor effects were observed on enzyme activity at different temperatures which were similar to some other species, such as *Brucella abortus* and *Schistosoma japonicum* [28, 61]. In addition, the most suitable pH value for rCpEno was 7.0–7.5, this result was consistent with previous studies showing that maximal enolase activity was observed at pH 7.4–7.6 in *P. falciparum* and pH 7.2 ± 0.1 in *T. gondii* [57, 60]. Compared with 0 mM salt ions (Ca^2+^, K^+^, Mg^2+^ and Na^+^), the enzyme activity was inhibited by increasing concentrations of metal ions (*P* < 0.05) while there was no obvious difference at 25 mM Mg^2+^ compared with 0 mM Mg^2+^ at the same time point (*P* > 0.05), which implied that low concentrations of Mg^2+^ (25 mM) had almost no effect on enzyme activity. This result was different with a study in *P. falciparum* by Pal-Bhowmick et al. [60] who reported the activity of *P. falciparum* enolase was inhibited by Na^+^, whereas K^+^ had a slight activating effect. They also found that low concentrations of Mg^2+^ had apparent activation effect and high concentrations had an inhibitory effect [60]. Previous research showed that Ca^2+^ was a “nonactivating” metal ion which inhibited the enzyme activity by replacing the catalytic Mg^2+^ ions [62]. In yeast enolase, two binding sites of Mg^2+^ have been reported, site I is a conformational site that could induce structural changes, and site II is a catalytic site that could promote catalytic activity [62, 63]. In *Trypanosoma brucei* enolase, another Mg^2+^ binding site had been reported, which was a low-affinity site involved in inhibition [64]. However, the function of Mg^2+^ binding sites in CpEno are still unclear, and there is a need for further crystal structure studies of CpEno. To our knowledge, this is the first report of the catalytic activity, reaction constant and effect factors for *C. parvum* enolase.

High titers of specific antibodies were induced after three immunisations with rCpEno, and good reactogenicity and immunogenicity were seen by ELISA and western blot. Using the antisera as a primary antibody, surface localisation of enolase on oocysts was revealed by fluorescence microscopy, which was consistent with previous studies in *Plasmodium* spp. where enolase located on the cell surface [36–38, 43]. However, in *E. tenalla* and *T. gondii*, enolases were also found in the nucleus [31, 49]. Due to the low number of fresh oocysts acquired for this research, the specific localisation of CpEno on sporozoites is still unclear. Further experiments on this aspect need to be further performed in the future.

Given the paucity of therapeutic drugs and the lack of effective vaccines, it is important to identify and characterise the specific surface molecules mediating attachment and invasion of the parasite into host cells [30]. Membrane proteins are involved in various critical biological processes [65], which are believed to mediate attachment and invasion of *Cryptosporidium*. In the present study, our first identification of CpEno on the surface by immunofluorescence examinations indicates that this protein may be a membrane-related protein. This was also confirmed in our laboratory when identifying membrane proteins of *C. parvum* sporozoites (unpublished). Therefore, along with a role as an essential glycolytic enzyme, CpEno may be involved in the attachment and/or invasion of IECs as in other organisms, including some apicomplexan protozoa. Previous studies have reported that enolase is one of the most frequent surface-localized glycolytic enzymes in many species, such as *Borrelia burgdorferi*, *G. duodenalis*, *Leishmania mexicana*, *Mycoplasma bovis*, *M. synoviae S. bovis*, *S. japonicum*, *Streptococcus pneumoniae*, *T. vaginalis* and *Vibrio parahaemolyticus* [12–17, 34, 52, 53, 61, 66]. In these pathogens, surface enolase plays an important role in host-pathogen interactions by binding plasminogen and is also possibly involved in pathogen invasion. In this study, a plasminogen binding assay showed that rCpEno could bind to human plasminogen, in agreement with studies on other species [12–17, 52, 53]. This suggests that rCpEno may participate as a parasitic virulence factor for *Cryptosporidium parvum*.

The publication of the *C. parvum* genome has enhanced our understanding on the parasite, but the function of a specific gene study was hindered for a long period in the past by the lack of a *C. parvum* long-term culture in vitro and a missing toolbox for genetic manipulation [67]. It is encouraging that the first system for genetic modification of *Cryptosporidium* was described, and a stable *C. parvum* transgenic line was established through a clustered regularly interspaced short palindromic repeat (CRISPR)/Cas9 system [68]. Using this system, research into the functions of enolase in *C. parvum* will be achieved through genetic manipulation and will help us deeply understand the role of enolase in the relationship between the parasite and host.

## Conclusions

In summary, a 1,350 bp enolase encoding gene was amplified from *C. parvum* cDNA and expressed in *E. coli* BL21 (DE3) to produce a recombinant protein rCpEno with a molecular weight of 52.5 kDa. Enzyme activity analysis showed that the reaction activity of rCpEno of converting 2-PGA to PEP was 33.5 U/mg and the most suitable pH value was 7.0–7.5. The *K*_*m*_ and *V*_*max*_ were 0.571 mM/l and 0.054 mM/l · min, respectively. The activities were inhibited with an increased concentration of metal ions, but minor effects were detected with temperature changes. Immunofluorescence assay showed that rCpEno localised on the surface of oocysts. The binding assay demonstrated that rCpEno could bind to human plasminogen with a dose-dependent pattern. To our knowledge, this is the first report describing the characteristics, binding activity and localisation of *C. parvum* enolase. The surface localisation of *C. parvum* enolase implied that it is not only a glycolytic enzyme but also participates in other processes of the parasite.

## Additional files

Additional file 1: Figure S1. Amplification of *cpeno* by RT-PCR. Lane 1: PCR product of *cpeno* gene amplified from *C. parvum* cDNA; Lane M: DL2,000 DNA Marker (Takara); Lane N: negative control. (TIF 325 kb)

Additional file 2: Figure S2. SDS-PAGE of rCpEno. Lane 1: uninduced pET28a-*cpeno* transfected *E. coli* BL21; Lane 2: induced pET28a-*cpeno* transfected *E. coli* BL21; Lanes 3 and 4: soluble and insoluble fractions of the induced pET28a-*cpeno* transfected *E. coli* BL21 extract, respectively; Lanes 5 and 6: purified rCpEno; Lane M: prestained protein ladder (Thermo Fisher Scientific). (TIF 670 kb)

Additional file 3: Figure S3. Western blot analysis of purified rCpEno. Purified rCpEno and induced pET-28a (+) plasmid transfected *E. coli* BL21 (DE3) competent cells were incubated with His-Tag mouse mAb (a) and *C. parvum* positive serum from cattle (b), respectively. Lane M: prestained protein ladder (Thermo Fisher Scientific). (TIF 902 kb)

## Abbreviations

2-PGA: 2-phosphoglycerate
BSA: Bovine serum albumin
CpEno: *C. parvum* enolase
*cpeno*: *C. parvum* enolase gene
CRISPR: Clustered regularly interspaced short palindromic repeat
DAB: 3,3’-diaminobenzidine
DAPI: 4’,6’-diamidino-2-phenylindole
ELISA: Enzyme-linked immunosorbent assay
FITC: Fluorescein isothiocyanate
HRP: Horseradish peroxidase
IECs: Intestinal epithelial cells
IPTG: Isopropyl-β-D-1-thiogalactopyranoside
*K*_*m*_: Michaelis constant
OD: Optical density
PBS: Phosphate buffer saline
PBST: Phosphate buffer saline (Tween 0.05%)
PCR: Polymerase chain reaction
PEP: Phosphoenolpyruvate
rCpEno: Recombinant *C. parvum* enolase
RT-PCR: Reverse transcription polymerase chain reaction
SDS-PAGE: Sodium dodecyl sulfate-polyacrylamide gel electrophoresis
TCA: Tricarboxylic acid
TMB: Tetramethylbenzidine
*V*_*max*_: Maximum reaction velocity
