# Supplementary figures and images for: Immunolocation and enzyme activity analysis of Cryptosporidium parvum enolase
Source: Parasit Vectors. 2017 May 31;10:273. doi: 10.1186/s13071-017-2200-y (PMC5452291; doi:10.1186/s13071-017-2200-y)

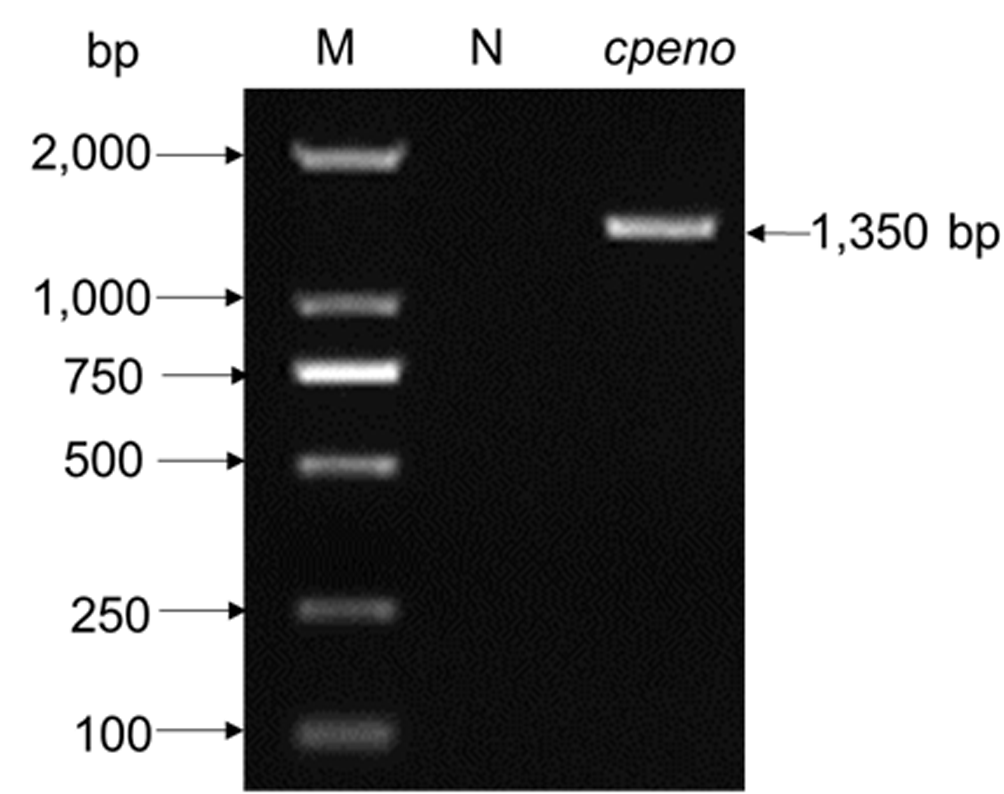

Supplement: Supplementary file 1 — Amplification of cpeno by RT-PCR. Lane 1: PCR product of cpeno gene amplified from C. parvum cDNA; Lane M: DL2,000 DNA Marker (Takara); Lane N: negative control. (TIF 325 kb) [file 13071_2017_2200_MOESM1_ESM.tif]

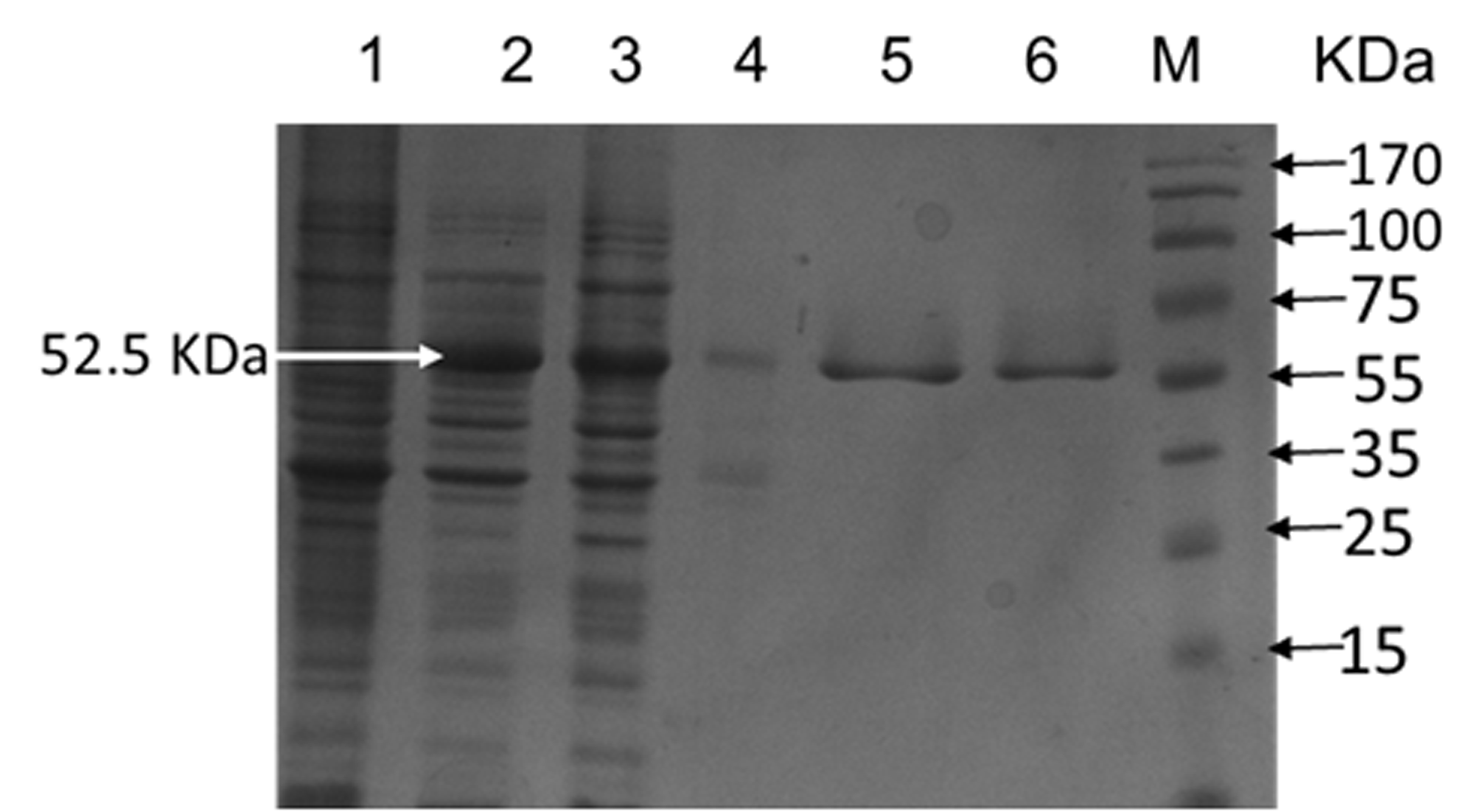

Supplement: Supplementary file 2 — SDS-PAGE of rCpEno. Lane 1: uninduced pET28a-cpeno transfected E. coli BL21; Lane 2: induced pET28a-cpeno transfected E. coli BL21; Lanes 3 and 4: soluble and insoluble fractions of the induced pET28a-cpeno transfected E. coli BL21 extract, respectively; Lanes 5 and 6: purified rCpEno; Lane M: prestained protein ladder (Thermo Fisher Scientific). (TIF 670 kb) [file 13071_2017_2200_MOESM2_ESM.tif]

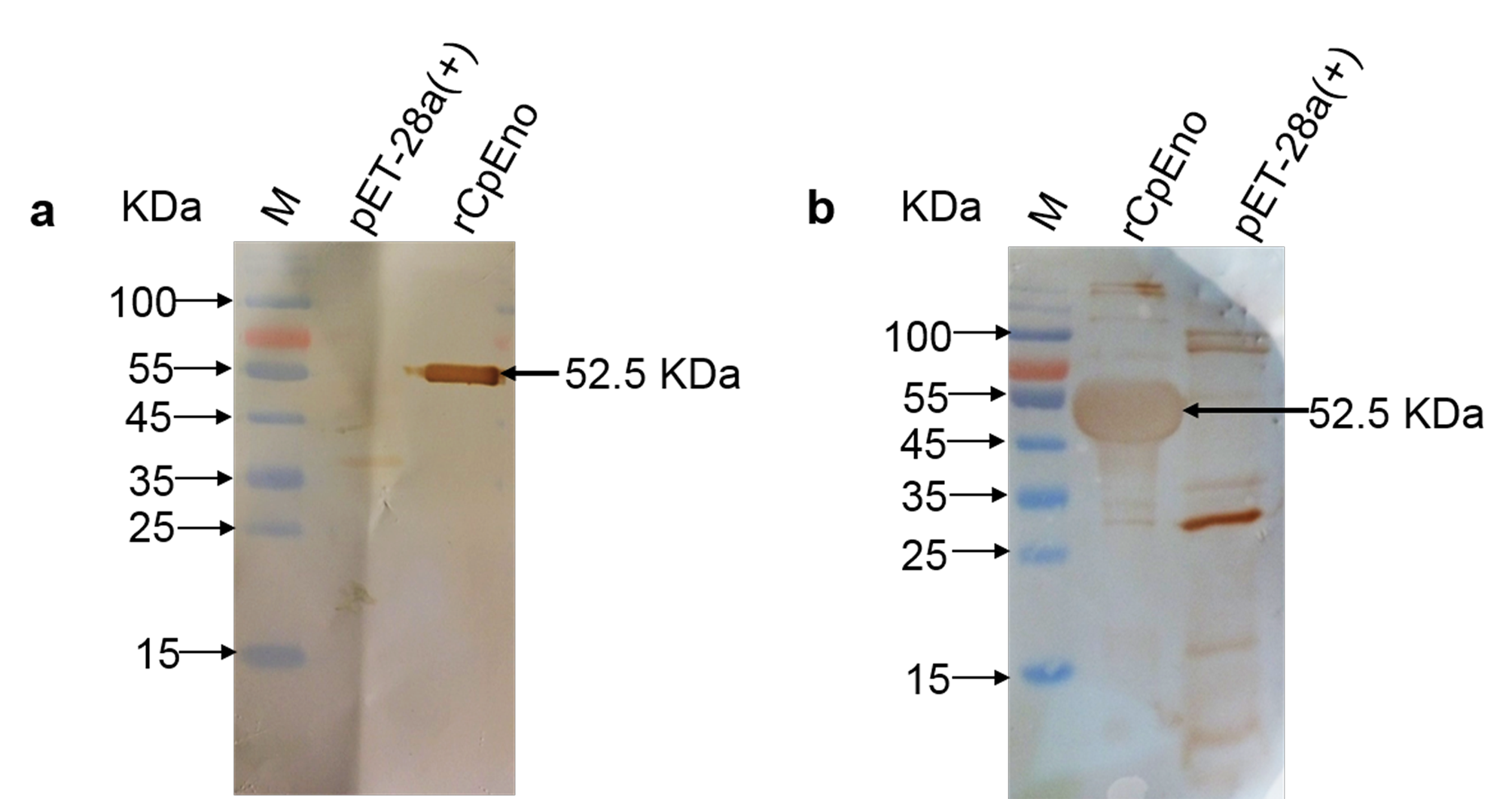

Supplement: Supplementary file 3 — Western blot analysis of purified rCpEno. Purified rCpEno and induced pET-28a (+) plasmid transfected E. coli BL21 (DE3) competent cells were incubated with His-Tag mouse mAb (a) and C. parvum positive serum from cattle (b), respectively. Lane M: prestained protein ladder (Thermo Fisher Scientific). (TIF 902 kb) [file 13071_2017_2200_MOESM3_ESM.tif]
